# Calcium Signaling in Oomycetes: An Evolutionary Perspective

**DOI:** 10.3389/fphys.2016.00123

**Published:** 2016-04-05

**Authors:** Limian Zheng, John J. Mackrill

**Affiliations:** Department of Physiology, BioSciences Institute, University College CorkCork, Ireland

**Keywords:** calcium channels, oomycetes, evolution, phylogenetics, inositol 1, 4, 5-trisphosphate receptors, ryanodine receptors

## Abstract

Oomycetes are a family of eukaryotic microbes that superficially resemble fungi, but which are phylogenetically distinct from them. These organisms cause major global economic losses to agriculture and fisheries, with representative pathogens being *Phytophthora infestans*, the cause of late potato blight and *Saprolegnia diclina*, the instigator of “cotton molds” in fish. As in all eukaryotes, cytoplasmic Ca^2+^ is a key second messenger in oomycetes, regulating life-cycle transitions, controlling motility and chemotaxis and, in excess, leading to cell-death. Despite this, little is known about the molecular mechanisms regulating cytoplasmic Ca^2+^ concentrations in these organisms. Consequently, this review analyzed the presence of candidate calcium channels encoded within the nine oomycete genomes that are currently available. This revealed key differences between oomycetes and other eukaryotes, in particular the expansion and loss of different channel families, and the presence of a phylum-specific group of proteins, termed the polycystic kidney disease tandem ryanodine receptor domain (PKDRR) channels.

## Roles of calcium signaling in oomycetes

**Figure 1:**
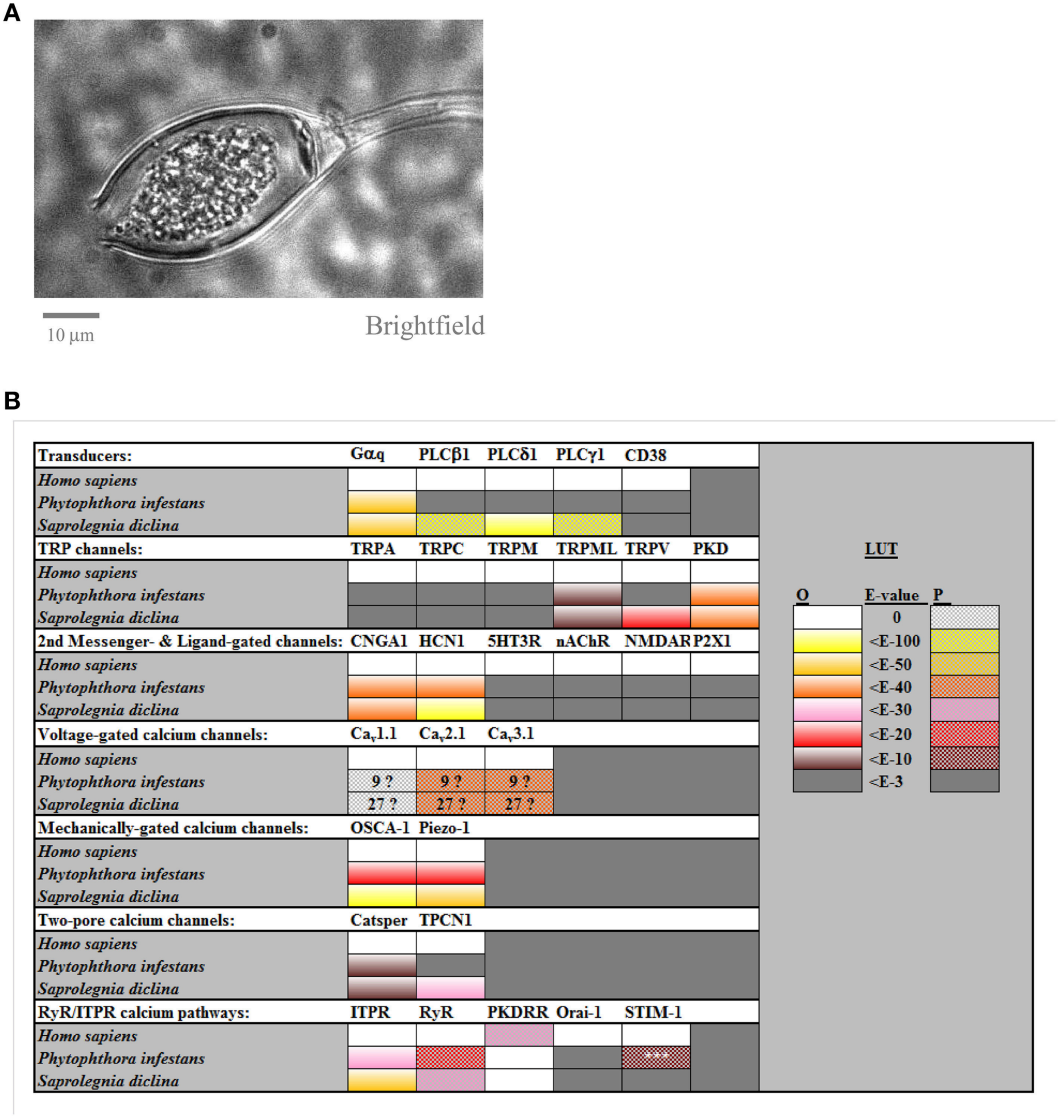
**Calcium channel families in ***Phytophthora infestans*** and in ***Saprolegnia diclina*****. Panel **(A)** shows a brightfield image of a cultured *P. infestans* sporangium, containing zoospores. Panel **(B)** compiles the results for BLAST searches for homolog of human calcium channels and transducers, carried out as described previously (Mackrill, 2012) and summarized from Supplemental Table 1. The LUT (look-up-table) gives a color code for the expected-values (*E*-values) for these searches, for either orthologs (“O”) or paralogs (“P”), taking 10^−3^ as the cut-off. The numbers in the boxes for voltage-gated calcium channels indicate the number of homologs found, due to uncertainty as to whether these were orthologs or paralogs. The homolog of STIM-1 found in *P. infestans, 1*, just shared a SAM motif with the search sequence and lacked other domains necessary for it to act as an ER calcium sensor.

Oomycetes are eukaryotic microbes that superficially resemble fungi, both in terms of their appearance and in the ecological niches which they occupy (Judelson, 2012). However, they are phylogenetically, physiologically, and biochemically distinct from fungi, being more closely related to diatoms and brown algae, with which they constitute the stramenopile kingdom (Verret et al., 2010). Oomycetes include members exhibiting parasitic or saprophytic lifestyles, with well-characterized examples including *Phytophthora infestans*, the instigator of late potato blight, and *Saprolegnia diclina* causing “cotton molds” in fish eggs, fry, and adults. Along with many other parasitic members, oomycete pathogens inflict multi-billion dollar losses per annum upon the agricultural and aquacultural sectors worldwide. Oomycetes have both vegetative and sexual life-cycles. They grow as mycelia, which upon appropriate stimulation, generate fruiting bodies called sporangia, see Figure 1A. These contain motile zoospores, whose release is triggered by specific cues and which migrate toward new hosts. Contact with a host leads to formation of a durable and adhesive cyst, which can subsequently germinate, and invade using an organ called the appressorium, can release secondary zoospores, or can persist in the encysted state. Given the crucial role of zoospores in the propagation of oomycetes, mechanisms controlling the development and biology of this life-cycle stage represent potential targets for the design of novel disease management strategies (Judelson and Blanco, 2005).

Calcium ions are utilized as a second messenger by all cellular organisms, controlling the majority of physiological processes in eukaryotes (Berridge et al., 2000; Plattner and Verkhratsky, 2013). The utility of Ca^2+^ in this role results from several factors. The first of these is that cells can rapidly alter cytoplasmic Ca^2+^ concentrations by the concerted actions of channels, pumps, and exchangers. Secondly, Ca^2+^ is heavily buffered by cellular components, allowing increases in Ca^2+^ to be either restricted to limited subcellular domains, or to spread throughout the cell if this buffering is overwhelmed. Thirdly, Ca^2+^ interacts with diverse effector proteins, which serve to translate changes in its concentration into biological responses. As is the case with most other eukaryotes, oomycetes contain multiple organelles that are potentially capable of storing and releasing Ca^2+^, including the endoplasmic reticulum (ER), Golgi apparatus, lysosomes, vesicles, and mitochondria (Chapman and Vujicic, 1965). In addition, they bear candidate Ca^2+^ handling organelles that are found within a narrower range of eukaryotic taxa, such as the vacuolar reticulum detected in the hyphal tips of various oomycetes including *Saprolegnia ferax* (Allaway et al., 1997), and the zoospore peripheral vesicles of *Phytophthora palmivora* (Irving et al., 1984). Like other eukaryotes, a large electrochemical gradient for Ca^2+^ exists across the plasma membrane (PM) of oomycetes, with cytoplasmic levels of about 100 nM in *Phytophthora cinnamomi* (Jackson and Hardham, 1996), relative to the high micromolar to low millimolar concentrations in the various extracellular media that these organisms encounter.

Among oomycetes, cytoplasmic Ca^2+^ has been demonstrated to regulate biological processes in every life-cycle stage. In *Achlya bisexualis*, the Ca^2+^ channel inhibitors verapamil, lanthanum and gadolinium all suppress the apical dominance of hyphal growth (Morris et al., 2011). Apical dominance is the process by which mycelial growth occurs mainly by extension of hyphal tips, rather than by generation of new branches. In *S. ferax*, calcium-sensitive microelectrodes have been used to detect a gradient of cytoplasmic Ca^2+^ within hyphae, with fluxes of this ion being greatest within 8 μm of the growing tip (Lew, 1999).

Cold-shock is a stimulus that triggers zoosporogenesis and zoospore release in many *Phytophthora* spp. In *P. cinnamomi*, this stimulus triggers a transient rise, followed by a sustained increase in cytoplasmic Ca^2+^, with the former phase being essential for cytokinesis and the formation of zoospores within sporangia (Jackson and Hardham, 1996). A comprehensive study in *P. infestans* revealed 15 genes whose transcription was enhanced during zoosporogenesis: for the majority of these genes, increased expression was abated by inhibitors of Ca^2+^ influx (verapamil, gadolinium), of the inositol 1,4,5-trisphosphate (IP_3_)-generating enzyme phospholipase C (PLC, blocked by U-73122), or of IP_3_ receptor/calcium release channels (ITPRs), antagonized by 2-aminoethoxydiphenyl borate (2-APB; Tani et al., 2004).

Zoospores migrate toward food sources using a range of stimuli to guide them, including chemotactic cues. Antisense mediated silencing of a Gα subunit-encoding gene in *P. sojae* severely compromised zoospore chemotaxis toward the soybean isoflavone daidzein (Hua et al., 2008). Such isoflavones also stimulate Ca^2+^ influx and increase the loading of intracellular Ca^2+^ stores in *P. sojae* zoospores (Connolly et al., 1999). The zoospores of several *Pythium* spp. typically swim in an extended helical pattern, with abrupt changes in direction on encountering noxious stimuli. Millimolar extracellular calcium causes the zoospores to swim in tight circles, whereas the Ca^2+^ chelator ethylene glycol tetraacetic acid (EGTA) elicited straight swimming (Donaldson and Deacon, 1993). Adhesion and germination of *Pythium aphaniderrnaturn* cysts was suppressed by EGTA and stimulated by Ca^2+^ (Donaldson and Deacon, 1992). In relation to the invasion of host tissues, appressorium formation by *P. palmivora* is suppressed by EGTA or gadolinium ions, but stimulated by the calcium ionophore A23187, indicating a requirement for elevated cytoplasmic Ca^2+^ in this process (Bircher and Hohl, 1999). In terms of sexual life-cycles, oosporogenesis in *Lagenidium giganteum* is suppressed by EGTA and promoted by a calcium ionophore (Kerwin and Washino, 1986).

## Calcium channel families in oomycetes

The preceding studies indicate fundamental roles for cytoplasmic Ca^2+^ in regulation of most aspects of oomycete biology. Consequently, the calcium channels potentially controlling cytoplasmic Ca^2+^ concentrations in these organisms represent key targets for the development of novel anti-oomycete “fungicides” for control of these pathogens. This is of paramount importance, given the development of resistance and changes in legislation for the use of such “fungicides” in several jurisdictions (Judelson and Blanco, 2005). Despite this, and the availability of several oomycete genomes, the calcium signaling mechanisms within these organisms have not been comprehensively surveyed. The remainder of this review aims to address this deficit, Figure 1B.

Homologs of human calcium signaling proteins were detected using BLASTP searches (Altschul et al., 1997) against oomycete genomes at the NCBI website (http://blast.ncbi.nlm.nih.gov/Blast.cgi?PROGRAM=blastp&PAGE_TYPE=BlastSearch&LINK_LOC=blasthome). The % identity, coverage and *E*-value of oomycete hits were recorded. The domain structures of these oomycete proteins were analyzed using the Conserved Domain Database tool at NCBI (Marchler-Bauer et al., 2015). In cases where the identity of oomycete proteins were ambiguous, they were searched back against the human proteome using BLASTP. The full genome databases of the two oomycete species analyzed most comprehensively in this study, *P. infestans* and *S. diclina*, were both generated at the Broad Institute and were of 8X and 171X coverage respectively. Consequently, it is unlikely that a lack of detection of oomycete homologs of human calcium signaling proteins in these searches is due to low genome quality.

### Transducers

In metazoa, extracellular stimuli such as hormones and neurotransmitters, acting by binding to specific receptors, are coupled to increases in cytoplasmic Ca^2+^ via the α-subunit of heterotrimeric G-proteins (Berridge et al., 2000). A paralog of *Homo sapiens* Gαq is encoded by all oomycete genomes examined, and plays a role in receptor-mediated, Ca^2+^-dependent chemotaxis of *P. sojae* zoospores (Connolly et al., 1999; Hua et al., 2008). In metazoa, receptors coupled to Gαq stimulate PLCβ isozymes, catalysing the breakdown of phosphatidylinositol-4,5-*bis*-phosphosphate to diacylglycerol and IP_3_, which gates ITPR calcium release channels in the ER. Of the other PLC families, PLCγ members are activated by receptor tyrosine kinases, whereas PLCδ enzynes are stimulated by increases in cytoplasmic Ca^2+^, thereby acting as a signal amplification mechanism. Although a homolog of *H. sapiens* PLCδ1 was detected in *S. diclina*, no PLC superfamily members were found in *P. infestans*, or in other non-saprolegnid oomycetes, Supplemental Table 1. This confirms a previous study (Meijer and Govers, 2006), and raises questions about how receptor activation of a G-protein stimulates a rise in Ca^2+^ in the absence of PLC. One possibility is the activation of PM Ca^2+^-influx channel‘s by the βγ-subunits of heterotrimeric G- proteins (Albert and Robillard, 2002). An alternative could be the activation of ADP-ribosyl cyclases, such as CD38, which generate a range of Ca^2+^-mobilizing second messengers including cyclic ADP ribose (cADPr) and nicotinic acid adenine dinucleotide diphosphate (NAADP; Ferrero et al., 2014). However, no homologs of *H. sapiens* CD38 were detected in *P. infestans* nor in *S. diclina*, Figure 1B.

### Transient receptor potential (TRP) channel superfamily

**Figure 2:**
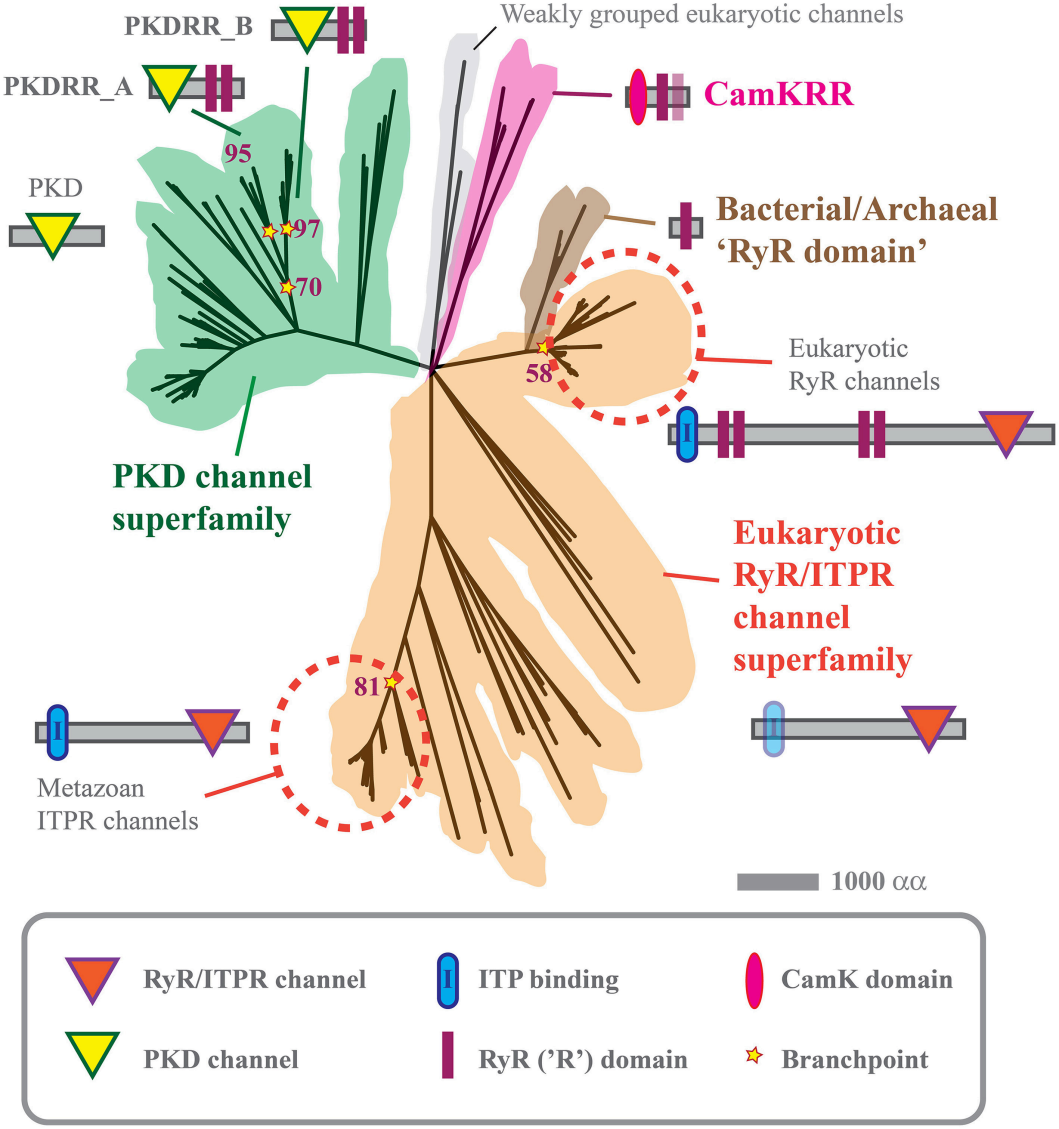
**Phylogenetic tree of PKD, CamKRR, and ITPR/RyR channel superfamilies**. Maximum likelihood tree constructed from the data given in Supplemental Table 2, using MEGA5 software, as described previously (Tamura et al., 2011; Mackrill, 2012). The tree represents a consensus of 500 bootstrap replicates and has a log likelihood of –93,088.11.

TRPs are a multigene superfamily of dimeric or tetrameric non-selective cation channels that can conduct Ca^2+^ (Verret et al., 2010; Nilius and Owsianik, 2011). They are gated by a variety of stimuli and are located in both the PM and in the membranes of organelles. Of the seven members of the TRP superfamily, homologs of TRPA (“ankyrin”), TRPC (“canonical”), TRPM (“melastatin”), and TRPN (“no mechanoreceptor potentials”, which is not found in mammals) were undetectable in oomycete genomes. Both *P. infestans* and *S. diclina* contain one homolog of human TRPML (“mucolipin”). Although, these TRPML homologs share low homology with vertebrate TRPML proteins, they display several canonical features, including a “PKD channel domain”, six strongly predicted transmembrane helices and an extended S1–S2 loop, Supplemental Table 1. Whereas the genome *S. diclina* encodes two TRPV (vanilloid) homologs, no members of this family were detectable in *P. infestans*. Both of the oomycete species analyzed contain multiple homologs of the polycystic kidney disease (PKD, TRPP, or “polycystin”) family, with seven members in *P. infestans* and nine in *S. diclina*, Figure 2.

### Second messenger- and ligand-gated calcium channels

These are gated by the binding of small molecule second messengers or extracellular ligands. Cyclic nucleotide gated channels (CNG) are activated by second messengers such as cAMP and cGMP. They are weakly voltage dependent and non-selective cation channels, which have been shown to be involved in Ca^2+^ signaling in diverse organisms, including animals and plants (Verret et al., 2010). Oomycete genomes encode multiple homologs of *H. sapiens* CNGA1 (up to 25 in *P. infestans* and 40 in *S. diclina*). However, some of these might not be CNGs, sharing identity only within cyclic nucleotide binding domains and lacking a recognizable channel-forming domain. Hyperpolarization-activated cation channels (HCN) also bind cyclic nucleotides, but are strongly activated at negative membrane potentials. They play important roles in generating rhythmic changes in membrane potential, as exemplified by the action potential in vertebrate hearts. Multiple HCN homologs were detected in oomycetes (21 in *P. infestans*, >50 in *S. diclina*), but many of these lack a discernible channel domains, although most possess candidate transmembrane helices (Supplemental Table 2) and share greatest identity within a putative kinase domain. Other ligand-gated calcium-permeable channel families, such as P2XR purinoceptors, inotropic glutamate receptors, 5HT-3 serotonin receptors, and nicotinic acetylcholine receptors (nAChR) are absent in oomycetes, Figure 1B and Supplemental Table 1.

### Voltage-gated and mechanically-gated calcium channels

Voltage-gated calcium channels (VGCC) open in response to membrane depolarization and evolved prior to the emergence of eukaryotes (Moran and Zakon, 2014; Stephens et al., 2015). They belong to a superfamily that includes voltage-gated sodium and potassium channels, and have a characteristic membrane topology of four repeats, each containing six transmembrane helices (Plattner and Verkhratsky, 2013; Moran and Zakon, 2014). The genomes of *P. infestans* and *S. diclina* encode multiple VGCC homologs (9 and 27 each), the selectivity filters of (Stephens et al., 2015) more closely resemble those of vertebrate VGCCs (“EEEE” and “SDEE”) than voltage-gated sodium or potassium channels Supplemental Data Figure 1 (Stephens et al., 2015). These candidate oomycete VGCCs are distinct from other channel families, such as yeast calcium channel 1 (CCH1) proteins. However, they lack an aspartic acid residue in domain II of their selectivity filters that is required for the calcium selectivity of vertebrate VGCCs, Supplemental Figure 1. Furthermore, no detectable homologs of the ancillary subunits of vertebrate VGCCs (β, α2/δ, γ) were encoded by the genomes of oomycetes, despite homologs of beta subunits being present in choanoflagellates (Moran and Zakon, 2014), eukaryotes that are more closely related to metazoans. However, antagonists of the dihydropyridine receptor family of VGCCs (Ca_v_1.x) have numerous effects on oomycete Ca^2+^ signaling (Donaldson and Deacon, 1993; Hill et al., 1998; Tani et al., 2004; Morris et al., 2011), indicating that certain members of this channel family are present, can act as calcium channels and have important roles in these organisms.

Mechanical stimuli play critical roles in oomycete life-cycles, in particular during zoospore release and encystment. Calcium channels that respond to such cues have been difficult to identify, even in well-characterized mammalian systems. Candidates include members of the TRP superfamily, osmotic stress activated calcium channels (OSCA; Yuan et al., 2014), and piezo mechanosensitive channels (Bagriantsev et al., 2014). *P. infestans* and *S. diclina* contain 1 or 3 homologs of *H. sapiens* piezo-1; and 8 or 9 homologs of human OSCA-1, suggesting that they possess multiple mechanisms with which to respond to mechanical stimuli.

### Two-pore calcium channels

CatSper and two-pore (TPCN) calcium channels form a superfamily of proteins that function as dimers, each of which contains two six-transmembrane domain repeats. TPCN channels are located within acidic organelles such as lysosomes, releasing Ca^2+^ in response to NAADP (Patel, 2015). Whereas *S. diclina* encodes five candidate homologs of human TPCN1, *P. infestans* shares only weak similarity with this protein, within features shared with VGCCs. More unexpectedly, both *P. sojae* and *P. ramorum* contain three TPCN homologs, which share greatest identity with *H. sapiens* TPCN2, in agreement with earlier work (Verret et al., 2010). This suggests a species-specific loss of TPCN calcium channels in *P. infestans*. Both *S. diclina* and *P. infestans* genomes contain multiple homologs of *H. sapiens* CatSper channels.

### Endoplasmic reticulum calcium channels

In mammalian cells, Ca^2+^ influx from the extracellular medium is essential for keeping intracellular calcium stores, such as the ER, replete. Maintenance of Ca^2+^ loading is achieved by interactions between ER Ca^2+^-sensors called STIM and PM channels of the Orai family (Park et al., 2009). No STIM/Orai homologs were detectable in any of the nine oomycete genomes analyzed, suggesting that these organisms use distinct mechanisms for maintaining the loading of their intracellular Ca^2+^ stores.

Such stores serve as a source of Ca^2+^ as a cytoplasmic second messenger, discharged by the gating of Ca^2+^-release channels, the two main families of which are members of the a common superfamily, the ITPRs and the ryanodine receptors (RyRs). Both are large (>2000 residue) multi-domain proteins, that function as tetrameric complexes. The current study indicates that *S. diclina* encodes 11 homologs of human ITPR1, seven of which contain a consensus IP_3_-binding domain, Figures 1, 2, Supplemental Data (Mackrill, 2012). In contrast, both *P. infestans* and *P. sojae* contain a single ITPR paralog, which lacks a discernible IP_3_-binding domain. This, along with the absence of PLC homologs (Meijer and Govers, 2006), Section on Transducers, brings into question the role of IP_3_-mediated Ca^2+^ signaling in *Phytophthora* spp., especially given the lack of selectivity of the pharmacological tools used to investigate this pathway.

RyRs have been identified in metazoan and sister groups, the choanoflagellates and *Capsaspora owczarzaki* (Mackrill, 2012; Plattner and Verkhratsky, 2013). These channels are pharmacologically gated by caffeine and this methyl xanthine supresses germination and promotes death in *P. infestans* sporangia (Hill et al., 1998). However, the only homologs of human RyR1 that were detected in *P. infestans* or *S. diclina* either more closely resembled ITPRs in terms of their protein domain organization, Figure 2, or only shared a high degree of identity at a so-called RyR (R)-domain. The alveolate *Paramecium tetraurelia* encodes an extensive array of calcium release channels within its genome. Some of these channels have domain structures that are intermediate between ITPRs and RyRs, indicating that there might have been a “blurring” of these channels families over evolutionary time (Plattner and Verkhratsky, 2013). It is possible that such evolutionary mechanisms could also explain the existence of a unique family of cation channels in oomycetes, which we have termed “PKDRR” channels.

### Polycystic kidney disease-RyR domain (PKDRR) channels

We have previously reported that both *P. infestans* and *S. diclina* each contain two candidate channel proteins which have a distinctive protein domain structure, not found in any other phylum (Mackrill, 2012). These contain a PKD channel domain [Section on Transient Receptor Potential (TRP) Channel Superfamily] in combination with a tandem repeat of RyR domains close to the C-terminus: hence, we have termed them PKDRR channels, although in many genome databases they are annotated as “ryanodine-inositol 1,4,5-triphosphate receptor Ca^2^ channel (RIR-CaC) family proteins.” The current analyses suggest that the latter descriptor is inaccurate: PKDRR proteins form a distinctive group within the PKD superfamily of channels (a total of seven members in *P. infestans* and nine in *S. diclina*), that are very distantly related to the RyR/ITPR superfamily, Figure 2. This phylogeny suggests that expansion and divergence of calcium release channels took place very early on during the evolution of eukaryotes.

PKDRR channels are encoded within all nine oomycete genomes analyzed, suggesting a conserved role, being maintained in the face of strong selection pressures (Judelson, 2012). Other oomycetes, such as *Aphanomyces* spp., contain up to four PKDRR homologs, Supplemental Table 2. The current phylogenetic analyses revealed that two subfamilies of these are conserved in oomycetes: PKDRR_A proteins have a PKD channel domain that is more N-terminal and a longer C-terminal tail after the last RyR domain, relative to members of PKDRR_B group, Figure 2.

RyR-domains are found multiple proteins in viruses, bacteria and archaea, but amongst eukaryotes have only been found in RyR and PKDRR channels, and in oomycete calmodulin-dependent protein kinases (CamK; Judelson and Roberts, 2002; Mackrill, 2012). Within near-atomic resolution structures rabbit RyR1, RyR domains act as sites of intra- and inter-molecular protein-protein interactions (Efremov et al., 2015). It is likely that these domains play similar roles in the other proteins which contain them, including oomycete PKDRR channels. RyR domains could have been incorporated into PKDRR proteins in a numbers of ways. They could have been present in a common ancestor of PKD channels and inherited vertically (Plattner and Verkhratsky, 2013); they might have been incorporated into pre-existing PKD channels by mosaic horizontal gene transfer from a prokaryotic organism (Mackrill, 2012); or they might have resulted from gene fusion between a PKD encoding and an RyR domain encoding gene within an ancestral oomycete genome. Oomycete genomes favor gene fusions, since they are diploid and their genes are “tightly packed” (Judelson, 2012). It is difficult to discern which mechanism is most likely, since these events probably took place very early on in eukaryotic history and traces of these events become less discernible with time. However, the genome of the oomycete *Albugo laibachii* bears some evidence of a gene fusion mechanism giving rise to these novel calcium channels: it contains a PKDRR protein (GenBank Acc. No. CCA17841.1) that is about twice as long as any other member and contains N- and C-terminal protein domains that are not found in any other member of this family.

## Perspectives on oomycete calcium signaling

Oomycetes have an extensive array of calcium channels, which presumably enable them to interact with their hosts in sophisticated ways. Certain families seem to have been lost in certain taxa, with the absence of detectable homolog of STIM/Orai, TRPA, TRPC, and TRPM being of note. In some cases, losses seem to be species-specific, with TPCN and TRPV being detected in *S. diclina*, but not in *P. infestans*. In other cases, calcium channels have expanded in oomycetes, with multiple voltage-gated and mechanically-gated cation channels contributing toward the sensory capabilities of these organisms. A highly conserved family of PKDRR channels is present in all oomycete genomes analyzed, but is absent from any other form of life. Given the conservation of this protein family in the face of strong selection pressures that occur in pathogen-host “arms races,” these channels presumably have a fundamental role in oomycete biology. Consequently, the PKDRR channels represent an attractive target for the development of oomycete-selective “fungicides.”

## Author contributions

JM conceived the idea for this mini-review; performed approximately half of the literature review and data collection; analyzed the data; and prepared the text and figures. LZ performed approximately half of the literature review and data collection; and critically reviewed several draft versions of the manuscript.

### Conflict of interest statement

The authors declare that the research was conducted in the absence of any commercial or financial relationships that could be construed as a potential conflict of interest.
