# Supplementary material for: Calcium Signaling in Oomycetes: An Evolutionary Perspective
Source: Front Physiol. 2016 Apr 5;7:123. doi: 10.3389/fphys.2016.00123 (PMC4820453; doi:10.3389/fphys.2016.00123)
Supplement: Supplementary file 1 [file Image1.PDF]

Supplementary Material

Calcium signalling in oomycetes: an evolutionary perspective

Limian Zheng, John James Mackrill*

* Correspondence: John Mackrill: j.mackrill@ucc.ie

Supplementary Figure 1. Alignment of the selectivity filters of human and oomycete voltage-gated cation channels.

Protein	Domain	Alignment	Position
H.sapiens Ca_v1.2	Domain I	CITMEGWTDV	359-368
H.sapiens Ca_v2.1	Domain I	CITMEGWTDL	312-321
H.sapiens Ca_v3.1	Domain I	VITLEGWVDI	347-356
H.sapiens Na_v1.4	Domain I	LMTQDYW-EN	401-410
P.infestans VGC	Domain I	SITQEGWTVI	376-385
S.diclina VGC	Domain I	IVTASGWMVL	379-388
		h	
H.sapiens Ca_v1.2	Domain II	ILTGEDWNSV	702-711
H.sapiens Ca_v2.1	Domain II	ILTGEDWNEV	663-672
H.sapiens Ca_v3.1	Domain II	ILTQEDWNKV	619-628
H.sapiens Na_v1.4	Domain II	ILCGE-IWET	756-764
P.infestans VGC	Domain II	IITGENWNSI	665-674
S.diclina VGC	Domain II	VITYDGYPNV	689-698
		hc	
H.sapiens Ca_v1.2	Domain III	VSTFEGWPEL	1331-1140
H.sapiens Ca_v2.1	Domain III	VSTGEGWPQV	1454-1463
H.sapiens Ca_v3.1	Domain III	LASKDGWVDI	1482-1491
H.sapiens Na_v1.4	Domain III	VATFKGWMDI	1240-1249
P.infestans VGC	Domain III	ISTTEGWADV	1136-1145
S.diclina VGC	Domain III	LTTLEGWIQV	1104-1113
		h	
H.sapiens Ca_v1.2	Domain IV	CATGEAWQDI	1460-1469
H.sapiens Ca_v2.1	Domain IV	SATGEAWHNI	1752-1763
H.sapiens Ca_v3.1	Domain IV	VSTGDNWNGI	1758-1769
H.sapiens Na_v1.4	Domain IV	ITTSAGWDGL	1532-1541
P.infestans VGC	Domain IV	AATGEAWNSC	1425-1434
S.diclina VGC	Domain IV	FSTGESWDNF	1390-1399
		h	

Full protein sequences of human Ca_v1.2 (Acc. No. NP_955630.3), Ca_v2.1 (Acc. No. NP_000059.3), Ca_v3.1 (Acc. No. NP_061496.2) and Na_v1.4 (Acc. No. NP_000325.4) were aligned against putative voltage-gated cation channels (VGC)from *P.infestans* (Acc. No. XP_002902845.1) and *S.diclina* (Acc. No. XP_008610030.1) using Clustal Omega software (http://www.ebi.ac.uk/Tools/msa/clustalo/). The positions of the high field strength (‘***h***’) and calcium-selectivity (‘***c***’) residues are indicated. *Magenta* shading indicates vertebrate voltage gated calcium channel-like; *blue*, vertebrate voltage gated sodium channel-like; and *green*, oomycete-like feature.
