# Supplementary material for: Calcium Signaling in Oomycetes: An Evolutionary Perspective
Source: Front Physiol. 2016 Apr 5;7:123. doi: 10.3389/fphys.2016.00123 (PMC4820453; doi:10.3389/fphys.2016.00123)
Supplement: Supplementary file 2 [file Table1.PDF]

Supplementary Material

Calcium signalling in oomycetes: an evolutionary perspective

Limian Zheng, John James Mackrill*
*** Correspondence: John Mackrill: j.mackrill@ucc.ie**

Supplementary Table 1: Oomycete homologues of human calcium signalling proteins

Species	Protein	%ID	E-value	Cover	Length	Homologs	Notes
	**Gαq**						
Homo sapiens	AAG61117.1	100	0.0E+00	100	359	1	4 other paralogues
Phytophthora infestans	XP_002906669.1	42	2.0E-89	96	355	1	
Saprolegnia diclina	XP_008609517.1	42	1.0E-88	96	354	1	
	**PLCβ1**						
Homo sapiens	Q9NQ66.1	100	0.0E+00	100	1216	4	
Phytophthora infestans	ND	ND	ND	ND	ND	ND	
Saprolegnia diclina	XP_008613806.1	36	7.0E-75	41	754	1	PLCδ-like when BLASTPed against *H.sapiens*
	**PLCδ1**						
Homo sapiens	NP_001124436.1	100	0.0E+00	100	777	4	
Phytophthora infestans	XP_002998121.1	24	2.0E-04	14	1069	0	C2-domain containing protein
Saprolegnia diclina	XP_008613806.1	35	6.0E-104	96	754	1	Homologues detected in other Saprolegniales (eg. *Aphanomyces sp* .), but not other oomycete orders
	**PLCg1**						
Homo sapiens	P19174.1	100	0.0E+00	100	1290	2	
Phytophthora infestans	XP_002896497.1	32	3.0E-04	5	162	0	SH3-domain containing protein
Saprolegnia diclina	XP_008613806.1	33	3.0E-48	49	754	1	PLCδ-like when BLASTPed against *H.sapiens*
	**CD38**						
Homo sapiens	NP_001766.2	100	0.0E+00	100	300	1	
Phytophthora infestans	ND	ND	ND	ND	ND	ND	
Saprolegnia diclina	ND	ND	ND	ND	ND	ND	
	**TRPA1**						
Homo sapiens	NP_015628.2	100	0.0E+00	100	1119	1	
Phytophthora infestans	XP_002998442.1	27	2.0E-37	47	870	0	Ankyrin domain containing kinase (and other Ank domain proteins)
Saprolegnia diclina	XP_008615426.1	27	2.0E-35	52	1185	0	Ankyrin domain containing kinase (and other Ank domain proteins)
	**TRPC1**						
Homo sapiens	P48995.1	100	0.0E+00	100	793	7	
Phytophthora infestans	ND	ND	ND	ND	ND	ND	
Saprolegnia diclina	ND	ND	ND	ND	ND	ND	
	**TRPM1**						
Homo sapiens	NP_002411.3	100	0.0E+00	100	1603	8	
Phytophthora infestans	ND	ND	ND	ND	ND	ND	
Saprolegnia diclina	ND	ND	ND	ND	ND	ND	
	**TRPML2**						
Homo sapiens	NP_694991.2	100	0.0E+00	100	566	3	
Phytophthora infestans	XP_008609330.1	20	5.0E-19	83	538	1	
Saprolegnia diclina	XP_002900700.1	22	2.0E-15	81	490	1	
	**TRPN**						
Drosophila melanogaster	NP_523483.2	100	0.0E+00	100	1761	1	
Phytophthora infestans	XP_002998442.1	31	2.0E-44	57	870	0	>50 Ankyrin binding domain proteins
Saprolegnia diclina	XP_008615426.1	29	3.0E-91	59	1185	0	>50 Ankyrin binding domain proteins
	**TRPV1**						
Homo sapiens	Q8NER1.2	100	0.0E+00	100	839	6	
Phytophthora infestans	XP_002906399.1	8	1.0E-05	32	145	0	>50 Ankyrin binding domain proteins
Saprolegnia diclina	XP_008618736.1	23	2.0E-24	77	773	2	(TRPV6-like)
	**PKD2 (TRPP)**						
Homo sapiens	NP_000288.1	100	0.0E+00	100	968	3	
Phytophthora infestans	XP_002904123.1	29	2.0E-40	33	924	7	Including 2 PKDRR
Saprolegnia diclina	XP_008608998.1	26	4.0E-41	52	654	9	Including 2 PKDRR
	**CNGA1**						
Homo sapiens	NP_001136036.1	100	0.0E+00	100	759	4	
Phytophthora infestans	XP_002899100.1	28	2.0E-46	67	708	25?	VIC superfamily members. Some might be CNGA: contain Crp domain
Saprolegnia diclina	XP_008614313.1	25	5.0E-41	59	623	40?	VIC superfamily members. Some might be CNGA: contain Crp domain
	**HCN1**						
Homo sapiens	AAO49469.1	100	0.0E+00	100	890	4	
Phytophthora infestans	XP_002899100.1	27	2.0E-49	52	708	21	Some are kinases, not channels
Saprolegnia diclina	XP_008613591.1	29	1.0E-56	54	1314	>50	> 50? Some are kinases, not channels
	**P2X1**						
Homo sapiens	P51575.1	100	0.0E+00	100	399	7	
Phytophthora infestans	ND	ND	ND	ND	ND	ND	
Saprolegnia diclina	ND	ND	ND	ND	ND	ND	
	**5-HT3R**						
Homo sapiens	P46098.1	100	0.0E+00	100	484	5	
Phytophthora infestans	ND	ND	ND	ND	ND	ND	
Saprolegnia diclina	ND	ND	ND	ND	ND	ND	
	**NMDA 1 isoform GluN1-1a**						
Homo sapiens	NP_015566.1	100	0.0E+00	100	938	7	
Phytophthora infestans	ND	ND	ND	ND	ND	ND	
Saprolegnia diclina	ND	ND	ND	ND	ND	ND	
	**nAChRα1**						
Homo sapiens	EAX11128.1	100	0.0E+00	100	457	10	
Phytophthora infestans	ND	ND	ND	ND	ND	ND	
Saprolegnia diclina	ND	ND	ND	ND	ND	ND	
	**Ca_v1.1**						
Homo sapiens	NP_000060.2	100	0.0E+00	100	1873	3	
Phytophthora infestans	XP_002902845.1	29	1.0E-155	78	1681	9?	VIC superfamily members; some probably VGCC; some probably Na^+ channels.
Saprolegnia diclina	XP_008610030.1	25	5.0E-131	79	1669	17?	
	**Ca_v2.1**						
Homo sapiens	NP_000059.3	100	0.0E+00	100	2266	3	
Phytophthora infestans	XP_002902845.1	30	3.0E-99	59	1681	9?	VIC superfamily members; some probably VGCC; some probably Na^+ channels.
Saprolegnia diclina	XP_008617256.1	24	2.0E-65	59	1669	17?	VIC superfamily members; some probably VGCC; some probably Na^+ channels.
	**Ca_v3.1**						
Homo sapiens	NP_061496.2	100	0.0E+00	100	2377	3	
Phytophthora infestans	XP_002902845.1	30	1.0E-86	50	1681	9?	VIC superfamily members; some probably VGCC; some probably Na^+ channels.
Saprolegnia diclina	XP_008603782.1	28	3.0E-63	43	1879	17?	VIC superfamily members; some probably VGCC; some probably Na^+ channels.
	**OSCA-1**						
Homo sapiens	XP_005249272.1	100	0.0E+00	100	772	2	
Phytophthora infestans	XP_005249272.1	22	7.0E-23	85	819	8?	
Saprolegnia diclina	XP_008620245.1	26	4.0E-63	87	792	9?	

	**Piezo-1**								
Homo sapiens	NP_001136336.2		100	0.0E+00	100	2521	2		
Phytophthora infestans	XP_002909616.1		24	2.0E-29	19	2774	1		
Saprolegnia diclina	XP_008608165.1		21	1.0E-34	33	2824	3		
	**CatSper1**								
Homo sapiens	Q8NEC5.3		100	0.0E+00	100	780	4		
Phytophthora infestans	XP_002902845.1		28	2.0E-13	22	1681	5		
Saprolegnia diclina	XP_008610029.1		27	3.0E-12	25	1445	6		
	**TPCN1**								
Homo sapiens	NP_001137291.1		100	0.0E+00	100	888	3		
Phytophthora infestans	XP_002904723.1		31	6.0E-12	18	500	6?	VIC and PIC families (NOT TPCN)	
Saprolegnia diclina	XP_008605512.1		27	8.0E-37	55	1429	5	TPCN family	
	**ITPR1**								
Homo sapiens	NP_001161744.1		100	0.0E+00	100	2743	3		
Phytophthora infestans	XP_002909590.1		32	2.0E-34	47	2916	1	RIH-RIH-RIHA domain structure, but no ITP BP or MIR domains.	ITPR1-like versus *H.sapiens* .
Saprolegnia diclina	XP_008613183.1		24	5.0E-65	42	2216	11	RIH-RIHA-C, no I or M. 7 of 11 have ITP BD	
	**RyR1**								
Homo sapiens	P21817.3		100	0.0E+00	100	5038	3		
Phytophthora infestans	XP_002909214.1		33	2.0E-30	10	1293	2	All PKDRR	
Saprolegnia diclina	XP_008607279.1		37	9.0E-32	14	1231	11	2 PKDRR	
	**Orai-1**								
Homo sapiens	Q96D31.2		100	0.0E+00	100	301	3		
Phytophthora infestans		ND	ND	ND	ND	ND	ND		
Saprolegnia diclina		ND	ND	ND	ND	ND	ND		
	**STIM-1**								
Homo sapiens	NP_001264890.1		100	0.0E+00	100	791	2		
Phytophthora infestans	XP_002902235.1		33	6.0E-04	11	1208	1?	SAM (Sterile Alpha Motif) domain	
Saprolegnia diclina		ND	ND	ND	ND	ND	ND		
