# Supplementary material for: Calcium Signaling in Oomycetes: An Evolutionary Perspective
Source: Front Physiol. 2016 Apr 5;7:123. doi: 10.3389/fphys.2016.00123 (PMC4820453; doi:10.3389/fphys.2016.00123)
Supplement: Supplementary file 3 [file Table2.PDF]

Supplementary Material

Calcium signalling in oomycetes: an evolutionary perspective

Limian Zheng, John James Mackrill*
*** Correspondence: John Mackrill: j.mackrill@ucc.ie**

Supplementary Table 2: Homologues of Oomycete PKDRR channels

Acc. No	Name	Species	Taxonomic Grouping	E-value	% ID	Length (αα)	Domain Structure	Notes
XP_002909214.1	Pinfestans_PKDRR1	*Phytophthora infestans*	Oomycetes	0	100	1293	PKD_R_R	2e-32/32% ID vs Hsapiens RyR2
XP_008901315.1	Pparasitica_PKDRR1	*Phytophthora parasitica*	Oomycetes	0	94	1334	PKD_R_R	
XP_009529392.1	Psojae_PKDRR1	*Phytophthora sojae*	Oomycetes	0	87	1305	PKD_R_R	PKD = PKD channel domain
CCA15061.1	Alaibachii_PKDRR1	*Albugo laibachii*	Oomycetes	0	53	1166	PKD_R_R	R = ryanodine receptor domain
CCI44400.1	Acandida_PKDRR1	*Albugo candida*	Oomycetes	0	53	1232	PKD_R_R	
XP_008611403.1	Sdiclina_PKDRR1	*Saprolegnia diclina*	Oomycetes	0	48	1341	PKD_R_R	
XP_012197723.1	Sparasitica_PKDRR1	*Saprolegnia parasitica*	Oomycetes	0	47	1318	PKD_R_R	
XP_008863215.1	Ainvadans_PKDRR1	*Aphanomyces invadans*	Oomycetes	0	43	1358	PKD_R_R	
XP_009843939.1	Aastaci_PKDRR1	*Aphanomyces astaci*	Oomycetes	0	42	1377	PKD_R_R	
XP_008872786.1	Ainvadans_PKDRR2	*Aphanomyces invadans*	Oomycetes	0	42	1245	PKD_R_R	
XP_009832416.1	Aastaci_PKDRR2	*Aphanomyces astaci*	Oomycetes	0	42	1245	PKD_R_R	
CCI49325.1	Acandida_PKDRR2	*Albugo candida*	Oomycetes	3.00E-70	26	1376	PKD_R_R	
CCA17841.1	Alaibachii_PKDRR2	*Albugo laibachii*	Oomycetes	1.00E-68	25	2549	GatB_PKD_R_R_SugarTrans	GatB = galactose binding
XP_002908895.1	Pinfestans_PKDRR2	*Phytophthora infestans*	Oomycetes	2.00E-47	27	1420	PKD_R_R	SugarTrans = sugar transporter
XP_009529777.1	Psojae_PKDRR2	*Phytophthora sojae*	Oomycetes	3.00E-46	28	1401	PKD_R_R	
XP_009835765.1	Aastaci_PKDRR3	*Aphanomyces astaci*	Oomycetes	1.00E-45	34	880	PKD_R_R	
XP_008868534.1	Ainvadans_PKDRR3	*Aphanomyces invadans*	Oomycetes	1.00E-45	34	1248	PKD_R_R	
XP_009835764.1	Aastaci_PKDRR4	*Aphanomyces astaci*	Oomycetes	4.00E-45	34	1251	PKD_R_R	
XP_012193958.1	Sparasitica_PKDRR2	*Saprolegnia parasitica*	Oomycetes	6.00E-45	34	1231	PKD_R_R	
XP_008607279.1	Sdiclina_PKDRR2	*Saprolegnia diclina*	Oomycetes	7.00E-45	34	1231	PKD_R_R	I = IP3 binding
ETI47942.1	Pparasitica_PKDRR2	*Phytophthora parasitica*	Oomycetes	9.00E-45	26	1386	PKD_R_R	M = MIR domain
XP_004342590.1	Cowczarzaki_RyR	*Capsaspora owczarzaki*	Ichthyosporea	1.00E-36	34	6625	I_M_H_S_S_R_R_H_H_R_R_A_C	H = ITPR/RyR homology doamin
CDS30480.1	Hmicrostoma_PKD	*Hymenolepis microstoma*	Platyhelminthes	1.00E-36	25	796	PKD	S = SPRY domain
XP_008758621.1	Rnorvegicus_PKD2L1	*Rattus norvegicus*	Chordata	3.00E-36	26	763	PKD	A = H associated domain
XP_005160146.2	Drerio_PKD2	*Danio rerio*	Chordata	3.00E-36	26	927	PKD	
KOF67457.1	Obimaculoides_PKD	*Octopus bimaculoides*	Lophotrochozoa	7.00E-36	26	780	PKD_EFh	EFh = EF-hand
XP_002119742.1	Cintestinalis_PKD2	*Ciona intestinalis*	Chordata	1.00E-35	24	887	PKD_EFh	
GAA37006.2	Csinensis_PKD2	*Clonorchis sinensis*	Platyhelminthes	3.00E-35	28	782	PKD	
XP_011975116.1	Mouton_PKD2	*Ovis aries musimon*	Chordata	6.00E-35	25	773	PKD	
XP_009954787.1	Ldiscolor_PKD2L1	*Leptosomus discolor*	Chordata	7.00E-35	26	844	PKD	
XP_013762742.1	Ttrahens_PKD2	*Thecamonas trahens*	Apusozoa	1.00E-34	24	832	PKD	
XP_006018170.1	Asinensis_PKD2	*Alligator sinensis*	Chordata	3.00E-34	26	765	PKD	
H2LRU7.2	Olatipes_PKD2	*Oryzias latipes*	Chordata	4.00E-34	25	901	PKD	
XP_012999134.1	Cporcellus_PKD2L1	*Cavia porcellus*	Chordata	5.00E-34	26	766	PKD	
XP_011194256.1	Bcucurbitae_PKD	*Bactrocera cucurbitae*	Ecdysozoa	6.00E-34	28	632	PKD	
XP_009303777.1	Drerio_RYR1	*Danio rerio*	Chordata	8.00E-34	35	5063	I_M_H_S_S_R_R_S_H_H_R_R_A_C	
NP_001246206.1	Dmelanogaster_RYR	*Drosophila melanogaster*	Ecdysozoa	1.00E-33	35	5115	I_M_H_S_S_R_R_S_H_H_R_R_A_C	
XP_012989794.1	Elucius_RYR1	*Esox lucius*	Chordata	2.00E-33	35	5076	I_M_H_S_S_R_R_S_H_H_R_R_A_C	
XP_004911138.1	Xenopus_PKD2	*Xenopus (Silurana) tropicalis*	Chordata	2.00E-33	26	946	PKD_EFh	
XP_008500272.1	Canna_PKD2L1	*Calypte anna*	Chordata	2.00E-33	25	838	PKD	
XP_009557836.1	Ccanorus_PKD2L1	*Cuculus canorus*	Chordata	2.00E-33	26	795	PKD_EFh	
CDQ69955.1	Omykiss_RYR1	*Oncorhynchus mykiss*	Chordata	3.00E-33	35	4791	I_M_H_S_S_R_R_S_H_H_R_R_A_C	
XP_007067623.1	Cmydas_PKD2L1	*Chelonia mydas*	Chordata	3.00E-33	26	853	PKD_EFh	
XP_005503700.1	Clivia_PKD2L2	*Columba livia*	Chordata	4.00E-33	26	648	PKD	
XP_005177530.1	Mdomestica_RYR	*Musca domestica*	Ecdysozoa	7.00E-33	35	5115	I_M_H_S_S_R_R_S_H_H_R_R_A_C	
XP_005805924.1	Xmaculatus_RYR1	*Xiphophorus maculatus*	Chordata	7.00E-33	35	5025	I_M_H_S_S_R_R_S_H_H_R_R_A_C	
XP_001657320.1	Aaegypti_RYR	*Aedes aegypti*	Ecdysozoa	9.00E-33	33	5118	I_M_H_S_S_R_R_S_H_H_R_R_A_C	
XP_013118743.1	Scalcitrans_RYR	*Stomoxys calcitrans*	Ecdysozoa	1.00E-32	34	5124	I_M_H_S_S_R_R_S_H_H_R_R_A_C	
XP_007437210.1	Pbivittatus_PKD2	*Python bivittatus*	Chordata	1.00E-32	27	763	PKD_EFh	
AHY02115.1	Bdorsalis_RYR	*Bactrocera dorsalis*	Ecdysozoa	2.00E-32	34	5140	I_M_H_S_S_R_R_S_H_H_R_R_A_C	
XP_007259518.1	Amexicanus_RYR1	*Astyanax mexicanus*	Chordata	2.00E-32	35	4720	I_M_H_S_S_R_R_S_H_H_R_R_A_C	
XP_012710791.1	Fheteroclitus_RYR1	*Fundulus heteroclitus*	Chordata	2.00E-32	35	5082	I_M_H_S_S_R_R_S_H_H_R_R_A_C	
XP_013127750.1	Oniloticus_RYR1	*Oreochromis niloticus*	Chordata	2.00E-32	35	5142	I_M_H_S_S_R_R_S_H_H_R_R_A_C	
XP_013766921.1	Pnyererei_RYR1	*Pundamilia nyererei*	Chordata	2.00E-32	37	5072	I_M_H_S_S_R_R_S_H_H_R_R_A_C	
XP_006800042.1	Nbrichardi_RYR1	*Neolamprologus brichardi*	Chordata	2.00E-32	37	5078	I_M_H_S_S_R_R_S_H_H_R_R_A_C	
XP_012778144.1	Mzebra_RYR1	*Maylandia zebra*	Chordata	2.00E-32	37	5090	I_M_H_S_S_R_R_S_H_H_R_R_A_C	
AAB58117.1	Mnigricans_RYR1	*Makaira nigricans*	Chordata	2.00E-32	37	5081	I_M_H_S_S_R_R_S_H_H_R_R_A_C	
XP_004527513.1	Ccapitata_RYR	*Ceratitis capitata*	Ecdysozoa	3.00E-32	34	5128	I_M_H_S_S_R_R_S_H_H_R_R_A_C	
XP_001370448.1	Mdomestica_PKD2	*Monodelphis domestica*	Chordata	3.00E-32	25	997	PKD_EFh	
XP_005285696.1	Cbellii_PKD2L1	*Chrysemys picta bellii*	Chordata	3.00E-32	26	853	PKD_EFh	
XP_007562016.1	Pformosa_PKD2	*Poecilia formosa*	Chordata	3.00E-32	26	891	PKD	
KDR20220.1	Znevadensis_PKD2	*Zootermopsis nevadensis*	Ecdysozoa	3.00E-32	23	611	PKD	
XP_013777532.1	Lpolyphemus_PKD2	*Limulus polyphemus*	Ecdysozoa	4.00E-32	27	716	PKD_EFh	
XP_011609906.1	Trubripes_RYR1	*Takifugu rubripes*	Chordata	5.00E-32	34	5080	I_M_H_S_S_R_R_S_H_H_R_R_A_C	
XP_007552444.1	Pformosa_RYR1	*Poecilia formosa*	Chordata	5.00E-32	36	5060	I_M_H_S_S_R_R_S_H_H_R_R_A_C	
XP_013178493.1	Pxuthus_RYR	*Papilio xuthus*	Ecdysozoa	5.00E-32	35	5136	I_M_H_S_S_R_R_S_H_H_R_R_A_C	
AKN21732.1	Smediterranea_PKD2L2	*Schmidtea mediterranea*	Platyhelminthes	6.00E-32	23	785	PKD_EFh	
AER25354.1	Pxylostella_RYR	*Plutella xylostella*	Ecdysozoa	6.00E-32	33	5117	I_M_H_S_S_R_R_S_H_H_R_R_A_C	
ETN61080.1	Adarlingi_RYR	*Anopheles darlingi*	Ecdysozoa	6.00E-32	33	5004	I_M_H_S_S_R_R_S_H_H_R_R_A_C	
XP_011420728.1	Cgigas_PKD2L1	*Crassostrea gigas*	Lophotrochozoa	7.00E-32	25	851	PKD_EFh	
KKF17507.1	Lcrocea_RYR2	*Larimichthys crocea*	Chordata	7.00E-32	36	5049	I_M_H_S_S_R_R_S_H_H_R_R_A_C	
XP_003742171.1	Moccidentalis_PKD2L	*Metaseiulus occidentalis*	Ecdysozoa	1.00E-31	25	781	PKD_EFh	
XP_008294801.1	Spartitus_RYR1	*Stegastes partitus*	Chordata	1.00E-31	37	5075	I_M_H_S_S_R_R_S_H_H_R_R_A_C	
XP_002718661.1	Ocuniculus_PKD2L1	*Oryctolagus cuniculus*	Chordata	1.00E-31	26	779	PKD	
XP_005795322.1	Xmaculatus_PKD2L1	*Xiphophorus maculatus*	Chordata	1.00E-31	26	753	PKD_DUF4546	DUF = domain of unknow function
XP_012408408.1	Sharrisii_PKD2	*Sarcophilus harrisii*	Chordata	1.00E-31	25	981	PKD_EFh	
XP_005932417.1	Hburtoni_PKD2	*Haplochromis burtoni*	Chordata	1.00E-31	25	902	PKD_EFh	
XP_012544761.1	Bmori_RYR	*Bombyx mori*	Ecdysozoa	1.00E-31	32	5123	I_M_H_S_S_R_R_S_H_H_R_R_A_C	
XP_009320668.1	Padeliae_PKD2L1	*Pygoscelis adeliae*	Chordata	1.00E-31	27	852	PKD_EFh	
XP_010122964.1	Cmacqueenii_PKD2L1	*Chlamydotis macqueenii*	Chordata	1.00E-31	26	852	PKD_EFh	
EFX89429.1	Dpulex_RYR	*Daphnia pulex*	Ecdysozoa	1.00E-31	35	5119	I_M_H_S_S_R_R_S_H_H_R_R_A_C	
XP_005444573.1	Fcherrug_PKD2	*Falco cherrug*	Chordata	2.00E-31	26	780	PKD_EFh_SOAR	STIM Orai1 activator region
XP_010154109.1	Ehelias_PKD2	*Eurypyga helias*	Chordata	2.00E-31	26	851	PKD_EFh	
XP_013814258.1	Amantelli_PKD2L1	*Apteryx australis mantelli*	Chordata	2.00E-31	26	852	PKD_EFh	
XP_013151587.1	Fperegrinus_PKD2	*Falco peregrinus*	Chordata	2.00E-31	26	753	PKD_EFh_SOAR	
KFB47317.1	Asinensis_RYR	*Anopheles sinensis*	Ecdysozoa	2.00E-31	33	5077	I_M_H_S_S_R_R_S_H_H_R_R_A_C	
XP_010561830.1	Hleucocephalus_PKD2L1	*Haliaeetus leucocephalus*	Chordata	2.00E-31	26	851	PKD_EF	
EEZ99829.1	Tcastaneum_RYR	*Tribolium castaneum*	Ecdysozoa	2.00E-31	33	4986	I_M_H_S_S_R_R_S_H_H_R_R_A_C	
XP_012679517.1	Charengus_RYR1	*Clupea harengus*	Chordata	3.00E-31	35	5072	I_M_H_S_S_R_R_S_H_H_R_R_A_C	
XP_003452262.1	Oniloticus_PKD2	*Oreochromis niloticus*	Chordata	3.00E-31	26	902	PKD_EFh	
XP_005025282.1	Aplatyrhynchos_PKD2	*Anas platyrhynchos*	Chordata	3.00E-31	26	852	PKD_EFh	
XP_013056948.1	Adomesticus_PKD2L1	*Anser cygnoides domesticus*	Chordata	4.00E-31	26	863	PKD_EFh	
XP_009943885.1	Ohoazin_PKD2L1	*Opisthocomus hoazin*	Chordata	4.00E-31	26	844	PKD_EFh	
AAI16298.1	Mmusculus_PKD2L1	*Mus musculus*	Chordata	4.00E-31	25	760	PKD_EFh	
XP_010410343.1	Ccornix_PKD2L1	*Corvus cornix cornix*	Chordata	4.00E-31	26	779	PKD_EFh_SOAR	
XP_011662382.1	Spurpuratus_PKD2L1	*Strongylocentrotus purpuratus*	Echinodermata	4.00E-31	24	907	PKD_EFh	
XP_010131522.1	Bsilvestris_PKD2L1	*Buceros rhinoceros silvestris*	Chordata	5.00E-31	26	844	PKD	
AKC03558.2	Csuppressalis_RYR	*Chilo suppressalis*	Ecdysozoa	5.00E-31	34	5133	I_M_H_S_S_R_R_S_H_H_R_R_A_C	
XP_008639484.1	Cbrachyrhynchos_PKD2	*Corvus brachyrhynchos*	Chordata	5.00E-31	26	762	PKD_EFh_SOAR	
ELT90189.1	Cteleta_PKD	*Capitella teleta*	Lophotrochozoa	4.00E-31	24	776	PKD_EFh	Edited search excluding oomycetes, chordata and ecdysozoa
BAB84714.1	Hpulcherrimus_RYR	*Hemicentrotus pulcherrimus*	Echinodermata	2.00E-30	34	5317	I_M_H_S_S_R_R_S_H_H_R_R_A_C	
CCD59582.1	Smansoni_RYR	*Schistosoma mansoni*	Platyhelminthes	4.00E-30	31	4998	I_M_H_S_S_R_R_S_H_H_R_R_A_C	
CEO98110.1	Pbrassicae_PKD	*Plasmodiophora brassicae*	Rhizaria	2.00E-29	25	702	PKD	
XP_013419430.1	Lanatina_PKD2	*Lingula anatina*	Lophotrochozoa	2.00E-29	24	852	PKD_EFh_OmpH	Outer membrane protein H
ELT95828.1	Cteleta_RYR	*Capitella teleta*	Lophotrochozoa	2.00E-29	31	5038	I_M_H_S_S_R_R_S_H_H_R_R_A_C	
XP_013386043.1	Lanatina_PKD1L2	*Lingula anatina*	Lophotrochozoa	6.00E-29	22	1225	GPS_PLAT_PKD	Latrophilin/CL-1-like GPS domain_PLAT/LH2 domain of polycystin-1 like proteins
CDS34291.1	Hmicrostoma_RYR	*Hymenolepis microstoma*	Platyhelminthes	6.00E-29	32	5717	I_M_H_S_S_R_R_S_H_H_R_R_A_C	
XP_009173549.1	Oviverrini_RYR	*Opisthorchis viverrini*	Platyhelminthes	8.00E-29	30	5727	I_M_H_S_S_R_R_S_H_H_R_R_A_C	
XP_005098719.2	Acalifornica_PKD2L1	*Aplysia californica*	Lophotrochozoa	5.00E-28	26	883	PKD_EFh	
XP_013090208.1	Bglabrata_PKD2L1	*Biomphalaria glabrata*	Lophotrochozoa	8.00E-28	26	698	PKD	
XP_009175902.1	Oviverrini_PKD	*Opisthorchis viverrini*	Platyhelminthes	1.00E-27	27	707	PKD	
XP_001639086.1	Nvectensis_PKD	*Nematostella vectensis*	Cnidaria	4.00E-27	26	903	PKD_EFh	
XP_009059256.1	Lgigantea_RYR	*Lottia gigantea*	Lophotrochozoa	5.00E-27	32	5045	I_M_H_S_S_R_R_S_H_H_R_R_A_C	
XP_009065053.1	Lgigantea_PKD	*Lottia gigantea*	Lophotrochozoa	6.00E-27	24	851	PKD_EFh	
XP_009164528.1	Oviverrini_PKDX	*Opisthorchis viverrini*	Platyhelminthes	2.00E-26	24	1581	GPS_PLAT_PKD	
XP_012793502.1	Shaematobium_PKD2L1	*Schistosoma haematobium*	Platyhelminthes	3.00E-26	28	720	PKD	
XP_013393030.1	Lanatina_RYR	*Lingula anatina*	Lophotrochozoa	4.00E-26	35	4715	I_M_H_S_S_R_R_S_H_H_R_R_A_C	
WP_033542974.1	Planococcus_R	*Planococcus sp. CAU13*	Firmicutes	8.00E-26	52	98	R	Bacterium
XP_002166908.2	Hvulgaris_PKD2L	*Hydra vulgaris*	Cnidaria	4.00E-25	24	684	PKD_OmpH	
WP_029326639.1	Bacillus_R	*Bacillus sp. m3-13*	Firmicutes	6.00E-25	56	95	R	Bacterium
CDD18636.1	Prevotella_R	*Prevotella sp. CAG:732*	Bacteroidetes	2.00E-24	53	95	R	Bacterium
XP_005830615.1	Gtheta_PKD	*Guillardia theta*	Cryptophyta	6.00E-24	25	749	PKD	
XP_006815503.1	Skowalevskii_PKD1L2	*Saccoglossus kowalevskii*	Hemichordata	7.00E-24	23	1233	PLAT_PKD	
WP_000323609.1	Bcereus_R	*Bacillus cereus*	Firmicutes	3.00E-23	54	94	R	CamK = calmodulin-dependent protein kinase

XP_002900460.1	Pinfestans_CamKRR	*Phytophthora infestans*	Oomycetes	2.00E-20	28	739	PH_CamK_R_R	Restricted search to P.infestans
XP_002904123.1	Pinfestans_PKD	*Phytophthora infestans*	Oomycetes	2.00E-15	26	924	PKD	
ETI46053.1	Pparasitica_CamKRR	*Phytophthora parasitica*	Oomycetes	0.E+00	88	732	PH_CamK_R_R	BLASTed Pinfestans_CamKRR
XP_009526058.1	Psojae_CamKRR	*Phytophthora sojae*	Oomycetes	0.00E+00	80	755	PH_CamK_R_R	
XP_008613197.1	Sdiclina_CamKRR	*Saprolegnia diclina*	Oomycetes	3.00E-139	37	692	PH_CamK_R_R	
XP_012208802.1	Sparasitica_CamKRR	*Saprolegnia parasitica*	Oomycetes	1.00E-132	38	820	PH_CamK_R_R	
XP_009832700.1	Aastaci_CamKRR	*Aphanomyces astaci*	Oomycetes	3.00E-126	34	727	PH_CamK_R_R	
CCA15407.1	Alaibachii_CamKR	*Albugo laibachii*	Oomycetes	4.00E-124	34	770	PH_CamK_R	Only 1 RyR domain
CCI48190.1	Acandida_CamKR	*Albugo candida*	Oomycetes	3.00E-123	33	770	PH_CamK_R	Only 1 RyR domain
XP_008871893.1	Ainvadans_CamKRR	*Aphanomyces invadans*	Oomycetes	6.00E-122	33	725	PH_CamK_R_R	
XP_002905287.1	Pinfestans_CamK	*Phytophthora infestans*	Oomycetes	7.00E-47	32	550	CamK	
ETK72490.1	Psojae_CamK	*Phytophthora parasitica*	Oomycetes	4.00E-46	33	551	CamK	
XP_009520373.1	Pparasitica_CamK	*Phytophthora sojae*	Oomycetes	2.00E-44	32	560	CamK	
XP_012194123.1	Sparasitica_CamK	*Saprolegnia parasitica*	Oomycetes	2.00E-41	31	471	CamK	
CCI42902.1	Acandida_CamK	*Albugo candida*	Oomycetes	4.00E-41	30	507	CamK	
XP_008864535.1	Ainvadans_CamK	*Aphanomyces invadans*	Oomycetes	1.00E-39	31	476	CamK	
XP_010937001.1	Eguineensis_CamK	*Elaeis guineensis*	Viridiplantae	5.00E-38	29	391	CamK	
CCA17689.1	Alaibachii_CamK	*Albugo laibachii*	Oomycetes	5.00E-38	29	504	CamK	
XP_008614206.1	Sdiclina_CamK	*Saprolegnia diclina*	Oomycetes	7.00E-38	34	511	CamK	
EIE77546.1	Rdelemar_CamK	*Rhizopus delemar*	Fungi	2.00E-37	31	446	CamK	
EWM25494.1	Ngaditana_CamK	*Nannochloropsis gaditana*	Eustigmatophyceae	1.00E-35	33	912	CamK	Non-oomycete Stramenopile
ETL46770.1	Pparasitica_PKD	*Phytophthora parasitica*	Oomycetes	0.00E+00	87	931	PKD	
XP_009537161.1	Psojae_PKD	*Phytophthora sojae*	Oomycetes	0.00E+00	82	938	PKD	
XP_012198419.1	Sparasitica_PKD	*Saprolegnia parasitica*	Oomycetes	3.00E-93	34	711	PKD	
XP_009842723.1	Aastaci_PKD	*Aphanomyces astaci*	Oomycetes	2.00E-92	30	754	PKD	
XP_008607890.1	Sdiclina _PKD	*Saprolegnia diclina*	Oomycetes	3.00E-92	32	711	PKD	
XP_008879364.1	Ainvadans_PKD	*Aphanomyces invadans*	Oomycetes	3.00E-84	81	762	PKD	
XP_008773755.1	Rnorvegicus_RYR3	*Rattus norvegicus*	Chordata	1.00E-30	32	4877	I_M_H_S_S_R_R_S_H_H_R_R_A_C	stats vs Pinfestans_PKDRR1
XP_012017113.1	Mouton_RYR3	*Ovis aries musimon*	Chordata	3.00E-30	31	4872	I_M_H_S_S_R_R_S_H_H_R_R_A_C	
XP_009293050.1	Drerio_RYR3	*Danio rerio*	Chordata	5.00E-31	31	4856	I_M_H_S_S_R_R_S_H_H_R_R_A_C	
XP_006018590.1	Asinensis_RYR3	*Alligator sinensis*	Chordata	5.00E-31	32	4872	I_M_H_S_S_R_R_S_H_H_R_R_A_C	
XP_011490347.1	Olatipes_RYR3	*Oryzias latipes*	Chordata	2.00E-31	31	4838	I_M_H_S_S_R_R_S_H_H_R_R_A_C	
XP_009558925.1	Ccanorus_RYR3	*Cuculus canorus*	Chordata	3.00E-31	32	4871	I_M_H_S_S_R_R_S_H_H_R_R_A_C	
XP_007235325.1	Amexicanus_RYR3	*Astyanax mexicanus*	Chordata	1.00E-31	31	4850	I_M_H_S_S_R_R_S_H_H_R_R_A_C	
NP_001076231.1	Ocuniculus_RYR3	*Oryctolagus cuniculus*	Chordata	3.00E-31	31	4872	I_M_H_S_S_R_R_S_H_H_R_R_A_C	
A2AGL3.1	Mmusculus_RYR3	*Mus musculus*	Chordata	1.00E-30	32	4863	I_M_H_S_S_R_R_S_H_H_R_R_A_C	
NP_114467.1	Rnorvegicus_RYR2	*Rattus norvegicus*	Chordata	5.00E-31	33	4953	I_M_H_S_S_R_R_S_H_H_R_R_A_C	
XP_011980519.1	Mouton_RYR2	*Ovis aries musimon*	Chordata	2.00E-31	33	4975	I_M_H_S_S_R_R_S_H_H_R_R_A_C	
XP_009291295.1	Drerio_RYR2	*Danio rerio*	Chordata	2.00E-26	29	4880	I_M_H_S_S_R_R_S_H_H_R_R_A_C	
XP_006016436.1	Asinensis_RYR2	*Alligator sinensis*	Chordata	1.00E-31	33	4956	I_M_H_S_S_R_R_S_H_H_R_R_A_C	
XP_011471845.1	Olatipes_RYR2	*Oryzias latipes*	Chordata	2.00E-32	33	4692	I_M_H_S_S_R_R_S_H_H_R_R_A_C	
XP_003473556.2	Cporcellus_RYR2	*Cavia porcellus*	Chordata	1.00E-31	33	4690	I_M_H_S_S_R_R_S_H_H_R_R_A_C	
XP_007069077.1	Cmydas_RYR2	*Chelonia mydas*	Chordata	1.00E-31	33	5011	I_M_H_S_S_R_R_S_H_H_R_R_A_C	
XP_013224527.1	Clivia_RYR2	*Columba livia*	Chordata	2.00E-31	33	4959	I_M_H_S_S_R_R_S_H_H_R_R_A_C	
XP_007244757.1	Amexicanus_RYR2	*Astyanax mexicanus*	Chordata	2.00E-26	28	4652	I_M_H_S_S_R_R_S_H_H_R_R_A_C	
P30957.3	Ocuniculus_RYR2	*Oryctolagus cuniculus*	Chordata	1.00E-31	33	4969	I_M_H_S_S_R_R_S_H_H_R_R_A_C	
XP_011242598.1	Mmusculus_RYR2	*Mus musculus*	Chordata	1.00E-30	33	4947	I_M_H_S_S_R_R_S_H_H_R_R_A_C	
XP_004993045.1	Srosetta_RYR	*Salpingoeca rosetta*	Choanoflagellida	3.00E-19	43	5340	I_H_S_R_S_H_H_R_R_C	
XP_002109077.1	Tadhaerens_RYR	*Trichoplax adhaerens*	Placozoa	3.00E-34	34	4949	I_M_H_S_R_R_S_H_H_R_R_A_C	
XP_002110052.1	Tadhaerens_PKD	*Trichoplax adhaerens*	Placozoa	3.00E-26	25	2734	WSC_REJ_GPS_PLAT_PKD	WSC domain; This domain may be involved in carbohydrate binding.<br>REJ domain; The REJ (Receptor for Egg Jelly) domain is found in PKD1
AAZ31363.1	Hsapiens_PKD2L2	*Homo sapiens*	Chordata	1.00E-31	25	758	PKD	
NP_057196.2	Hsapiens_PKD2L1	*Homo sapiens*	Chordata	4.00E-31	25	805	PKD	
XP_006711866.1	Hsapiens_RYR2	*Homo sapiens*	Chordata	2.00E-30	32	4984	I_M_H_S_S_R_R_S_H_H_R_R_A_C	
XP_011520182.1	Hsapiens_RYR3	*Homo sapiens*	Chordata	3.00E-30	32	4859	I_M_H_S_S_R_R_S_H_H_R_R_A_C	
XP_006723382.1	Hsapiens_RYR1	*Homo sapiens*	Chordata	4.00E-30	33	5027	I_M_H_S_S_R_R_S_H_H_R_R_A_C	
EWM27927.1	Ngaditana_PKD	*Nannochloropsis gaditana*	Eustigmatophyceae	7.00E-07	28	718	PKD	Stramenopiles without Oomycetes
CEM13223.1	Vbrassicaformis_PKD	*Vitrella brassicaformis*	Alveolata	2.00E-33	24	937	PKD	Alveolates
EJY78633.1	Otrifallax_PKD	*Oxytricha trifallax*	Alveolata	3.00E-19	28	1598	PKD_PKD	Alveolates
CDW80624.1	Slemnae_PKD2L1	*Stylonychia lemnae*	Alveolata	2.00E-18	26	1624	PKD_PKD	Alveolates
XP_013252140.1	Eacervulina_PKD	*Eimeria acervulina*	Alveolata	1.00E-11	24	1502	PKD	Alveolates
XP_001022905.2	Tthermophila_PKD	*Tetrahymena thermophila*	Alveolata	1.00E-10	20	1469	TctB	Alveolates
ITPR homologs with identity to Hsapiens_ITPR1, searched with Pinfestans_ITPR1								
XP_002909590.1	Pinfestans_ITPR1	*Phytophthora infestans*	Oomycetes	0.00E+00	100	2916	H_H_A	
XP_009514398.1	Psojae_ITPR1	*Phytophthora sojae*	Oomycetes	0.00E+00	68	3001	H_H_A	
ETL44115.1	Pparasitica_ITPR1	*Phytophthora parasitica*	Oomycetes	0.00E+00	84	3789	I_M_H_H_A_FAM117	
XP_008878887.1	Ainvadans_ITPR1	*Aphanomyces invadans*	Oomycetes	0.00E+00	37	3037	I_M_H_A	
XP_012197373.1	Sparasitica_ITPR1	*Saprolegnia parasitica*	Oomycetes	0.00E+00	39	1873	A_C	
XP_009839519.1	Aastaci_ITPR1	*Aphanomyces astaci*	Oomycetes	0.00E+00	37	3199	I_M_H_A_PHA	
XP_008620853.1	Sdiclina_ITPR1	*Saprolegnia diclina*	Oomycetes	3.00E-113	37	2913	I_M_H_A_C	
XP_008613183.1	Sdiclina_ITPR2	*Saprolegnia diclina*	Oomycetes	2.00E-101	35	2216	H_A_C	
XP_009843560.1	Aastaci_ITPR2	*Aphanomyces astaci*	Oomycetes	6.00E-98	34	2845	I_M_H_A	
XP_008880167.1	Ainvadans_ITPR2	*Aphanomyces invadans*	Oomycetes	3.00E-93	34	2655	I_M_H_A_C	
CDW78570.1	Slemnae_ITPR1	*Stylonychia lemnae*	Alveolata	4.00E-69	26	2894	I_M_H_H_A_C	
XP_008610948.1	Sdiclina_ITPR3	*Saprolegnia diclina*	Oomycetes	9.00E-64	35	2786	I_M_A_C	
XP_001017190.2	Tthermophila_ITPR1	*Tetrahymena thermophila*	Alveolata	6.00E-63	24	2827	H_H_A_C	
EJY80876.1	Otrifallax_ITPR1	*Oxytricha trifallax*	Alveolata	1.00E-61	24	2724	I_H_H_A_C	
KOO21485.1	Chrysochromulina_ITPR	*Chrysochromulina sp. CCMP291*	Haptophyceae	6.00E-57	24	3272	I_H_A_C	
XP_001014415.2	Tthermophila_ITPR2	*Tetrahymena thermophila*	Alveolata	9.00E-56	24	2860	I_H_H_A_C	
XP_008861937.1	Ainvadans_ITPR3	*Aphanomyces invadans*	Oomycetes	2.00E-55	27	2697	I_H_H_A_C	
XP_009827762.1	Aastaci_ITPR3	*Aphanomyces astaci*	Oomycetes	2.00E-55	27	2703	I_H_H_A_C	
XP_009827764.1	Aastaci_ITPR4	*Aphanomyces astaci*	Oomycetes	3.00E-55	27	1933	H_A_C	
XP_008618486.1	Sdiclina_ITPR4	*Saprolegnia diclina*	Oomycetes	6.00E-54	38	2441	I_H_A_C	
CDW83968.1	Slemnae_ITPR2	*Stylonychia lemnae*	Alveolata	5.00E-53	27	2773	I_M_H_H_A_C	
XP_009827523.1	Aastaci_ITPR5	*Aphanomyces astaci*	Oomycetes	3.00E-53	24	2841	I_A_C	
EJY68355.1	Otrifallax_ITPR2	*Oxytricha trifallax*	Alveolata	2.00E-51	26	2877	I_H_H_A_C	
XP_004333542.1	Acastellanii_ITPR	*Acanthamoeba castellanii*	Amoebozoa	1.00E-49	37	3344	I_H_H_A_C	
XP_004347577.1	Cowczarzaki_ITPR	*Capsaspora owczarzaki*	Ichthyosporea	2.00E-49	36	3232	I_M_H_H_A_C	
XP_011605455.1	Trubripes_ITPR2	*Takifugu rubripes*	Chordata	6.00E-49	36	2679	I_M_H_H_A_C	
XP_008861941.1	Ainvadans_ITPR4	*Aphanomyces invadans*	Oomycetes	1.00E-47	27	2665	I_H_H_A	
CCC21095.1	Ptetraurelia_TPA_I_1a	*Paramecium tetraurelia*	Alveolata	2.00E-46	34	2972	I_H_A_C	
XP_014646869.1	Csimum_ITPR2	*Ceratotherium simum simum*	Chordata	3.00E-46	37	2670	I_M_H_H_A_C	
XP_007453130.1	Lvexillifer_ITPR1	*Lipotes vexillifer*	Chordata	7.00E-46	33	2744	I_M_H_H_A_C	
XP_003762438.1	Sharrisii_ITPR1	*Sarcophilus harrisii*	Chordata	9.00E-46	33	2743	I_M_H_H_A_C	
XP_007500194.1	Mdomestica_ITPR1	*Monodelphis domestica*	Chordata	9.00E-46	33	2750	I_M_H_H_A_C	
XP_009300863.1	Drerio_ITPR1	*Danio rerio*	Chordata	2.00E-45	34	2683	I_M_H_H_A_C	
XP_008271820.1	Ocuniculus_ITPR1	*Oryctolagus cuniculus*	Chordata	2.00E-45	32	2725	I_M_H_H_A_C	
AAB04947.2	Hsapiens_ITPR1	*Homo sapiens*	Chordata	4.00E-45	32	2710	I_M_H_H_A_C	
XP_012817182.1	Xtropicalis_ITPR1	*Xenopus (Silurana) tropicalis*	Chordata	5.00E-45	35	2702	I_M_H_H_A_C	
AIU40167.1	Tcastaneum_ITPR	*Tribolium castaneum*	Ecdysozoa	1.00E-44	35	2724	I_M_H_H_A_C	
XP_013804517.1	Aaustralis_ITPR1	*Apteryx australis mantelli*	Chordata	2.00E-44	32	2750	I_M_H_H_A_C	
CCC21096.1	Ptetraurelia_TPA_I_1c	*Paramecium tetraurelia*	Alveolata	2.00E-44	34	2980	I_M_H_A_C	
AGN03925.1	Djaponica_ITPR	*Dugesia japonica*	Platyhelminthes	2.00E-44	39	2666	I_M_H_H_A_C	
XP_014460900.1	Amississippiensis_ITPR1	*Alligator mississippiensis*	Chordata	2.00E-44	32	2644	I_M_H_H_A_C	
XP_006808860.1	Nbrichardi_ITPR1	*Neolamprologus brichardi*	Chordata	7.00E-44	33	2744	I_M_H_H_A_C	
XP_008166289.1	Cbelli_ITPR1	*Chrysemys picta bellii*	Chordata	8.00E-44	32	2718	I_M_H_H_A_C	
XP_013775375.1	Lpolyphemus_ITPR	*Limulus polyphemus*	Ecdysozoa	3.00E-43	34	2765	I_M_H_H_A_C-Abhyd	
XP_007907205.1	Cmilii_ITPR2	*Callorhinchus milii*	Chordata	4.00E-43	34	2694	I_M_H_H_A_Ydil	
BAA05384.1	Hsapiens_ITPR2	*Homo sapiens*	Chordata	8.00E-43	33	2701	I_M_H_H_A_C	
KRY53102.1	Tbritovi_ITPR	*Trichinella britovi*	Ecdysozoa	6.00E-41	33	2663	I_M_H_H_A_C	
XP_004938060.1	Ggallus_ITPR2	*Gallus gallus*	Chordata	8.00E-41	33	2717	I_M_H_H_A_C	
NP_001300740.1	Drerio_ITPR3	*Danio rerio*	Chordata	8.00E-40	33	2634	I_M_H_H_A_C	
XP_002939518.3	Xtropicalis_ITPR3	*Xenopus (Silurana) tropicalis*	Chordata	1.00E-37	34	2673	I_M_H_H_A_PAH	
EMP32350.1	Cmyda_ITPR3	*Chelonia mydas*	Chordata	2.00E-38	35	2570	I_M_H_H_A_C	
XP_011620146.1	Trubripes_ITPR3	*Takifugu rubripes*	Chordata	4.00E-36	33	2682	I_M_H_H_A	
XP_001747685.1	Mbrevicollis_ITPR	*Monosiga brevicollis*	Choanoflagellida	6.00E-36	30	2669	I_M_H_H_A_C	
XP_004649843.1	Jjaculus_ITPR3	*Jaculus jaculus*	Chordata	1.00E-35	33	2795	I_M_H_H_A_Coli	
